# Preeruptive Intracoronal Radiolucencies: Detection and Nine Years Monitoring with a Series of Dental Radiographs

**DOI:** 10.1155/2017/6261407

**Published:** 2017-11-27

**Authors:** Chanika Manmontri, Phattaranant May Mahasantipiya, Papimon Chompu-inwai

**Affiliations:** ^1^Division of Pediatric Dentistry, Department of Orthodontics and Pediatric Dentistry, Chiang Mai University, Chiang Mai, Thailand; ^2^Division of Oral Radiology, Department of Oral Biology and Diagnostic Sciences, Chiang Mai University, Chiang Mai, Thailand

## Abstract

Preeruptive intracoronal radiolucencies (PEIRs) are mostly incidentally found by routine radiographic examination of unerupted teeth. PEIRs are classified into two types according to the nature of the lesion: progressive and nonprogressive. A case report of a 17-year-old boy with a nonprogressive PEIR on the permanent mandibular left second molar is presented. The lesion was initially detected on an unerupted tooth at age eight years, eight months. It was clinically and radiographically assessed yearly. Cone beam computed tomography (CBCT) was used to evaluate the lesion's size and location when the patient was 11 and 14 years old. The assessments confirmed that the lesion was nonprogressive and had no connection to the pulp or oral cavity. Due to the static nature of the detected PEIR during the nine-year follow-up period, the patient's low caries-risk status, and high patient and parental cooperation in periodic dental care, it was decided to place resin sealant on the affected tooth and monitor the lesion without any operative treatment.

## 1. Introduction

Preeruptive intracoronal radiolucency (PEIR), also called preeruptive intracoronal dentin defect or preeruptive intracoronal resorption, is often incidentally found on various types of commonly prescribed radiographs, including panoramic [1–8], periapical [9–11], and bitewing [1, 4, 9, 10, 12–15] radiographs. Typical radiographic characteristics of PEIR are radiolucent lesions similar to dental caries or coronal resorption in unerupted teeth of both primary [3, 16] and permanent [1–15] dentitions. The lesions are often located within the coronal dentin, adjacent to the dentino-enamel junction (DEJ), and are found as single or multiple lesions in both maxillary and mandibular arches [2, 4, 17–19]. Clinically, most PEIR-affected teeth have no defects or lesions on the outer enamel surface, and no remarkable difference is found between affected and contralateral teeth [1–4, 8–11, 13–15]. Proposed etiologies include acquired pathological conditions from apical inflammation of predecessor primary teeth or dental caries of the unerupted teeth and developmental defect of dentin due to localized defective mineralization or invasion of resorptive cells during crown formation [4, 7, 9, 10]. As most histological case studies have found that PEIR lesions consist of inflammatory resorptive cells without evidence of microbial invasion, dental caries, or pulp degeneration, the hypothesis that PEIR is caused by resorption is now mostly accepted [4].

PEIR has been reported in various countries with a subject prevalence of 0.7%–27.3% and tooth prevalence of 0.5%–3.47% [18–26]. No association has been found between PEIR and sex, race, medical conditions, systemic factors, or fluoride supplementation. Highly caries-prone occlusal surfaces in young individuals often result in occlusal caries, which, once communication with a preexisting PEIR lesion occurs, may result in a disastrous outcome [9]. Therefore, early detection, before eruption, should be achieved from dental radiographs commonly prescribed for other purposes. Early detection can also lead to timely appropriate management. Clinicians should be aware of PEIR and carefully inspect for PEIR in every unerupted tooth appearing on the child's radiographs.

PEIR has been classified, according to its progressive nature, into two major types: nonprogressive (static) [4, 12, 13, 27] or progressive (developing) [3, 5, 6, 10, 14, 28]. Treatment is usually provided according to the type of the lesion. In a nonprogressive case, many case reports support the more conservative approach by waiting until the tooth has erupted into the oral cavity before treatment [12, 13]. The rationales behind this protocol are the asymptomatic nature of affected teeth and the static nature of the lesion. Accordingly, this group of authors proposes that the dental practitioner should wait and monitor the progression of the lesion by periodic radiographic examination. Whenever the lesion increases in size, treatment should be provided immediately [12, 13].

On the contrary, in some case reports, the lesions progressed in size [3, 5, 6, 10, 14, 28]. These reports retrospectively examined the radiographs recorded before discovery of the lesions and found that PEIRs were smaller or not present in previous radiographs. Furthermore, some case reports presented severe cases of PEIRs which were extensive, progressing rapidly, or symptomatic [29–31]. Consequently, not only early detection of PEIR but also determining the nature of the lesion is critical for treatment planning [24]. After the first detection of PEIR on unerupted teeth, the dental practitioner can use periodic radiographic examination to classify the lesion as progressive or nonprogressive to aid in making a proper treatment plan for each lesion [24].

Because the lesion can only be detected, monitored, and differentiated through dental radiographs, the benefits of several types of dental imaging are demonstrated in this case report. The purposes of this case report were to (1) promote awareness and early detection of PEIR through commonly prescribed dental radiographs; (2) describe the typical characteristics of PEIR; (3) highlight the importance of periodic examination that is necessary for differentiation of the types of the lesion; and (4) make use of advanced diagnostic imaging in confirming the characteristics of the lesion.

## 2. Case Presentation

**Figure 1:**
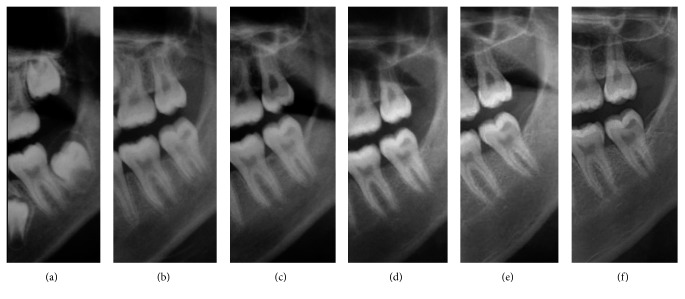
Cropped panoramic radiographs demonstrating a PEIR-affected tooth (permanent mandibular left second molar) when the patient was (a) 8 years, 8 months old, (b) 10 years, 11 months old, (c) 12 years, 5 months old, (d) 13 years, 4 months old, (e) 15 years, 3 months old, and (f) 17 years, 4 months old. There was no obvious progression of PEIR size or changes in the PEIR location noted from this film series.

PEIR on the unerupted permanent mandibular left second molar was first incidentally discovered from the panoramic radiograph (Planmeca 2002 cc Proline, Planmeca, Helsinki, Finland), prescribed for interceptive orthodontic purposes, of a healthy eight-year, eight-month-old Thai boy (Figure 1). He was free of systemic diseases or congenital syndromes. The tooth was not in the ectopic position. The lesion was located in the distal part of the crown and appeared as a radiolucent band under the DEJ. Since the tooth was unerupted and only the crown had formed, the treatment plan was to monitor the lesion by using intraoral and extraoral radiographs periodically to determine lesion progression and tooth development.

**Figure 2:**
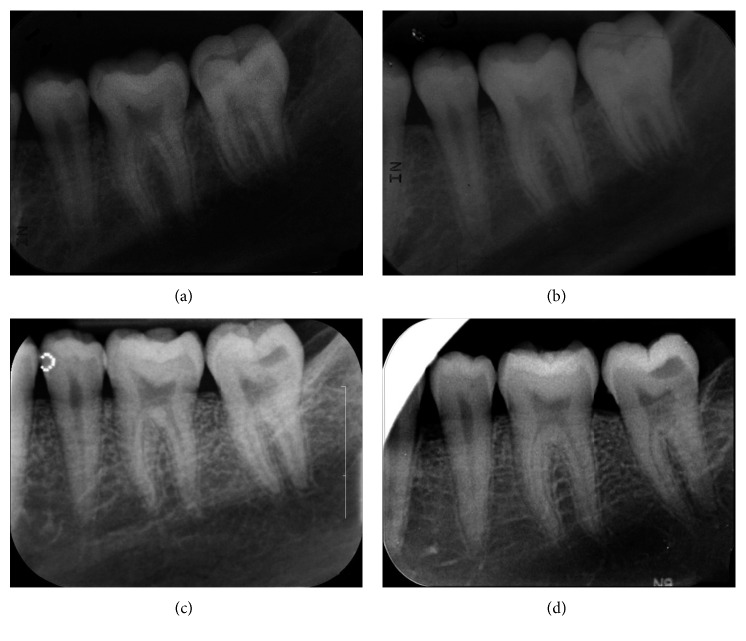
Periapical radiographs of the PEIR-affected tooth (permanent mandibular left second molar) when the patient was (a) 10 years, 11 months old, (b) 11 years, 5 months old, (c) 15 years, 3 months old, and (d) 17 years, 4 months old. The tooth is in the normal path of eruption, has normal crown formation, and has had continuing root formation since the first radiograph.

When the patient was 10 years, 11 months old, the PEIR-affected tooth was partially erupted and asymptomatic and had no abnormalities, caries, or enamel defects on its occlusal surface. The panoramic and periapical radiographs (Figures 1 and 2) showed that approximately two-thirds of the root length had developed. The PEIR was still present at the same location as previously described. The PEIR size was slightly larger on the latter than the earlier radiographs. However, there were no signs of pulpal involvement and no tooth formation abnormalities. Consequently, no treatment was rendered at that time.

**Figure 3:**
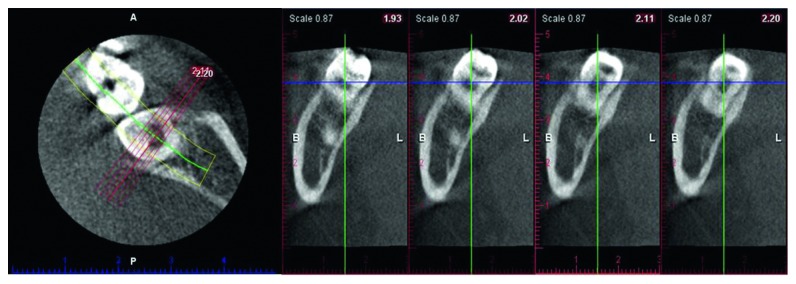
Sagittal slices of the PEIR-affected tooth (permanent mandibular left second molar) reconstructed from the first CBCT when the patient was 11 years, 5 months old. PEIR presented as an obvious radiolucency within the dentin with no direct exposure of the lesion or dentin to the oral cavity. The distance from the midfloor of the lesion to the roof of the pulpal area was 1.58 mm. The PEIR dimension is 3.75 mm occluso-gingival depth, 5.52 mm bucco-lingual width, and 3.05 mm mesio-distal length.

When the patient was 11 years, five months old, the PEIR-affected tooth was still partially erupted, symptom-free, and without any clinical signs of coronal defects. A periapical radiograph (Figure 2) did not reveal the lesion clearly due to superimposition of the PEIR and the anterior border of the ramus. From all the previous film series, the location, size, and relationship of the PEIR lesion to other structures could not be clearly identified. Consequently, cone beam computed tomography (CBCT) (Planmeca Promax 3D, Planmeca) was further used to reaffirm the characteristics of the lesion (Figure 3). Due to the small size and the closed system of the lesion determined by CBCT, the tooth was planned to be continually followed up.

**Figure 4:**
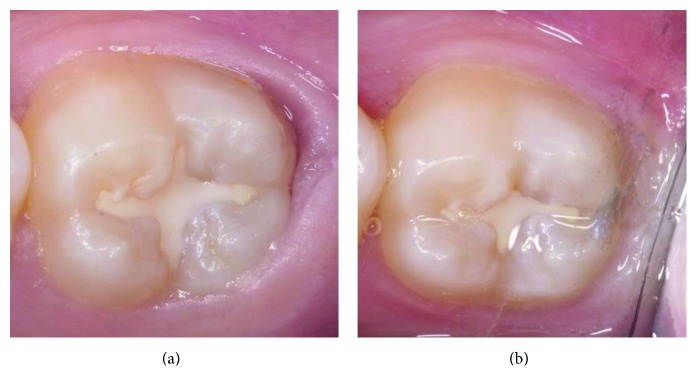
Occlusal appearance of the PEIR-affected tooth (permanent mandibular left second molar) (a) after sealant placement when the patient was 12 years, 5 months old and (b) at a follow-up visit when the patient was 14 years, 9 months old.

At the age of 12 years, five months, the affected tooth was completely erupted revealing a similar appearance to the mirror images of the contralateral tooth. The affected tooth had intact enamel and normal occlusal features with deep pits and fissures. Resin sealant (Concise Light Cure White Sealant, 3M ESPE, St. Paul, MN, USA) was placed on the affected tooth and on the other permanent second molars for caries prevention (Figure 4). Particular attention in sealing deep pits and fissures must be paid to teeth with PEIR because communication of occlusal caries and the existing PEIR lesion may result in severe damage to tooth structure.

**Figure 5:**
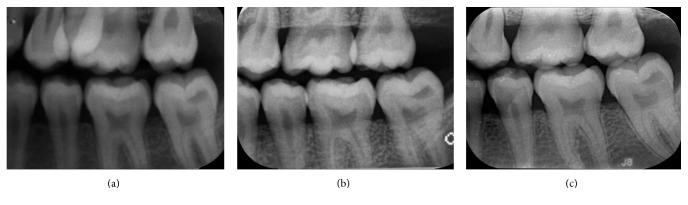
Bitewing radiographs of the PEIR-affected tooth (permanent mandibular left second molar) when the patient was (a) 13 years, 4 months old, (b) 15 years, 3 months old, and (c) 17 years, 4 months old. The PEIR has no connection to the enamel or the pulp of the affected tooth.

**Figure 6:**
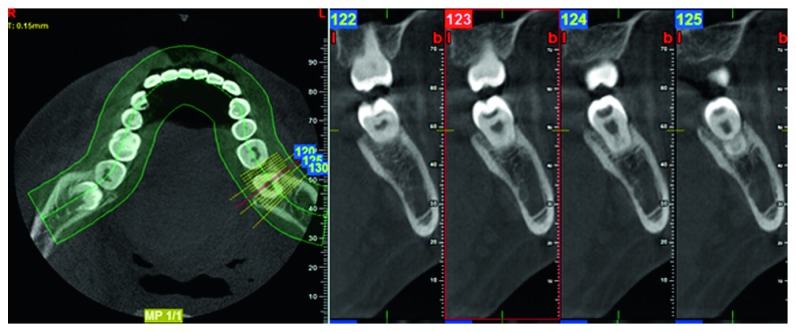
Sagittal slices of the PEIR-affected tooth (permanent mandibular left second molar) reconstructed from the second CBCT when the patient was 14 years, 9 months old. The PEIR location was reconfirmed to be just under the dentino-enamel junction and not connected to the pulp chamber or oral environment. The distance between the midfloor of the lesion and the pulpal area is 2.00 mm. The PEIR dimension is 2.30 mm occluso-gingival depth, 4.00 mm bucco-lingual width, and 3.40 mm mesio-distal length.

The affected tooth was reevaluated clinically and radiographically twice yearly until the patient was 14 years, nine months old (Figures 1, 1, 4, and 5). The clinical and radiographic findings from these follow-up visits confirmed that the patient continued to be absent of any abnormal signs or symptoms related to the PEIR-affected tooth, the tooth presented positive results to electric pulp test and cold test, and the PEIR remained relatively the same size and in relatively the same position as in the previous radiographs. To confirm the size, location, and invasion of the lesion to surrounding structures, CBCT (NewTom VGi, NewTom, Verona, Italy) (Figure 6) was repeated three years later. Unfortunately, a different CBCT machine was used unavoidably at this visit because the previously used machine in our institution had been replaced. Consequently, the results could not be directly compared to the previous results. However, the CBCT results reconfirmed that the lesion was small and had no connection to the pulp chamber or oral environment.

Due to the nonprogressive nature of the reported lesion, minimally invasive dentistry was selected as the treatment of choice for this case. The dentist, parents, and patient were in consensus to avoid any operative treatments until any abnormal signs or symptoms occurred. Because of the patient's age and low caries-risk status, he has been recalled every 6 to 12 months, and has had radiographic examination of the lesion every 12 months (Figures 1, 2, and 5). The latest recall appointment before this report was when he was 17 years, four months old. There were no changes clinically or radiographically in the PEIR-affected tooth (Figures 1, 2, and 5).

## 3. Discussion

The PEIR presented in this case report shared similar clinical and radiographic characteristics of PEIR described in the mandibular second molar in previous case reports [1–3, 9, 10, 12, 13]. Clinically, the patients in those reports had no symptoms, and the affected teeth had no defects on the outer enamel surface and no remarkable difference to the contralateral tooth. Radiographically, the PEIR lesions presented as a radiolucent, globular, or hemispherical lesion presenting in the dentin under the DEJ without a capsule or penetration into the enamel. The enamel thickness above the lesion and the dentin appearance of the affected tooth were of the same quality and quantity as in the contralateral tooth. Also, there was no connection between the lesion and the pulp.

We noticed that panoramic examinations provided comparable diagnostic information to the intraoral radiographs in terms of the lesion's position and its correlation to surrounding structures. Although intraoral radiographs generally provide more accuracy than do panoramic radiographs, their accuracy was sometimes compromised from unobtainable adequate film positioning for the paralleling technique in this young patient. Consequently, clinicians should carefully examine all unerupted teeth from the prescribed radiographs for the presence of PEIR. If PEIR is detected, either intraoral or extraoral radiographs should be adequate for monitoring the lesion. Decisions about radiographic type should be based on various factors, for example, age, cooperation, and caries risk of the patient, including the need for radiographs for other oral problems, so that unnecessary, repeated radiation exposure can be avoided. Based on this case, 6 to 12 month intervals for evaluation of the PEIR were sufficient to diagnose the lesion as a static type and to confirm that the lesion did not compromise the development of the affected tooth. However, there have been no established recommendations of periodicity of PEIR radiographic evaluation.

Both intraoral and extraoral traditional radiographs have some limitations, especially considering that only two-dimensional information is given. To evaluate the dimensional change in the PEIR lesion, these radiographs may be insensitive [4]. Therefore, CBCT was used twice in a three-year interval to give more precise three-dimensional information, including size, location, and the relation of the PEIR lesion to surrounding structures, and confirmed that the PEIR on the affected tooth was nonprogressive. To the best of our knowledge, this case report is the first to use a series of CBCT images to confirm the characteristics of PEIR and has the longest follow-up period without any intervention to the lesion.

During a nine-year follow-up with clinical and radiographic examination, the detected PEIR was confirmed to be static. The longitudinal radiographs provided in our case report support the theory that the progressive resorption of PEIR may cease or decelerate after tooth eruption, possibly due to discontinuation of the vascular supply from the surrounding external tissues of the crown [1, 8, 15], because the lesion size in our case had not changed since the tooth erupted.

We propose that PEIR should not always be treated with invasive treatment, such as surgical exposure, operative treatment, or extraction. Because the PEIR lesion in our case was considered to be nonprogressive, we decided to follow the protocol of delayed restoration [1, 4]. Nevertheless, both patient and parents were aware of the susceptibility to fracture of the undermined enamel, which may cause the affected tooth to require restoration later [4]. However, earlier restorative intervention may also put the tooth at risk of fracture, secondary caries, restoration failure, or pulpal symptoms, which may also jeopardize the longevity of this tooth [32]. We believed that resin sealants with proper maintenance and preventive measures would prevent the patient from developing any carious lesions in the affected tooth. Czarnecki et al. [11] speculated that proper timing of sealant placement could be performed either preeruptively or posteruptively. The case presented here had sealant placement posteruptively, and the tooth was monitored without any restorative treatment for 59 months. To avoid the affected tooth from being through restorative cycles, sealant placement is considered to be a logical approach in this nonprogressive case.

## 4. Conclusions

Investigating the images of all unerupted teeth on a radiograph is the key to early detection of PEIR lesions. With thorough information from clinical and radiographic examinations, dental practitioners can provide proper management of the lesion at the appropriate time. The progressive nature of the lesion, caries risk, and follow-up compliance of the patient should be considered as factors for treatment planning. If the lesion is nonprogressive in low caries-risk patients with good compliance, minimal invasive dentistry is preferable, as demonstrated in this case.
